# Influence of Neighborhood Disparities on Traumatic Shoulder Instability Severity and Timing of Care in Adolescents

**DOI:** 10.1177/03635465251346901

**Published:** 2025-06-23

**Authors:** Dang-Huy Do, John E. Arvesen, James J. McGinley, Amareesa K. Robinson, Eliza E. Lovrich, Henry B. Ellis, Philip L. Wilson

**Affiliations:** † Department of Orthopaedic Surgery, University of Texas Southwestern Medical Center, Dallas, Texas, USA; ‡ Scottish Rite for Children Orthopedic and Sports Medicine Center, Frisco, Texas, USA; Investigation performed at the Scottish Rite for Children Orthopedic and Sports Medicine Center, Frisco, Texas, USA

**Keywords:** shoulder instability, pediatric sports medicine, health disparity, socioeconomic

## Abstract

**Background:**

Identifying and understanding socioeconomic disparities among adolescents with traumatic shoulder instability can help to optimize care for patients by improving differences in the disease burden, disease severity, and awareness of resource limitations. Current studies evaluating disparities among patients with shoulder instability are limited to the adult population or surgical patients.

**Purpose:**

To evaluate how educational, health/environmental, and social/economic disparities influence the timing of shoulder instability treatment and shoulder instability severity among adolescents.

**Study Design:**

Cohort study; Level of evidence, 3.

**Methods:**

A retrospective review of patients aged 10 to 19 years diagnosed with shoulder instability from January 2022 to April 2024 at a single institution was conducted. The Child Opportunity Index (COI) was used to evaluate inequalities in educational, health/environmental, and social/economic opportunities. Disease severity was determined using magnetic resonance imaging (MRI), including glenoid bone loss, Hill-Sachs interval size, distance to dislocation, and presence of an off-track lesion. Continuous variables were analyzed with the Mann-Whitney *U* test or the Spearman correlation coefficient, while categorical variables were analyzed using the chi-square test. Significance was set at *P* < .05.

**Results:**

There were 181 patients who met the inclusion criteria. Patients with a lower overall COI had a longer time from injury to initial presentation (*r* = −0.15; *P* = .048), injury to orthopaedic evaluation (*r* = −0.17; *P* = .027), and injury to MRI (*r* = −0.16; *P* = .033) but not from injury to surgery. A history of recurrent dislocations was associated with a lower overall COI (B = −3.27; *P* = .041), lower educational COI (B = −3.01; *P* = .009), and lower social/economic COI (B = −3.65; *P* = .049). Patients with a distance to dislocation <10 mm were associated with a lower overall COI (B = −7.59; *P* = .003), lower educational COI (B = −8.38; *P* = .045), lower health/environmental COI (B = −7.88; *P* = .006), and lower social/economic COI (B = −8.22; *P* = .001).

**Conclusion:**

Children living in neighborhoods with fewer educational and social/economic opportunities were associated with longer times from injury to orthopaedic evaluation and from injury to MRI for shoulder instability and were at a higher risk for recurrent shoulder dislocations.

Children living in disadvantaged neighborhoods with a lower socioeconomic status are known to have delays in care and clinical outcomes for orthopaedic diseases and injuries.^2,37^ These children have been shown to have greater delays from injury to diagnosis and treatment, missed postoperative appointments, have a longer time to fracture union, and have worse patient-reported outcomes.^12,19,33,41^ There has been a strong emphasis on understanding and addressing health care disparities over recent years. Among children, social determinants of health extend to the family and include education level, food security, transportation needs, housing stability, and financial strain.^2,37^

A shoulder dislocation occurs at a rate of 23.9 per 100,000 person-years, with 40% of those occurring in patients aged 10 to 19 years.^44^ This particular age group is especially at risk for recurrent dislocations, even after arthroscopic soft tissue stabilization.^4,34^ Timely treatment is important to prevent future instability events and associated secondary soft tissue and bony injuries.^10,38^ However, only 50% of patients who present to the emergency room with a first-time anterior shoulder dislocation follow up with a specialist.^22^ Among those who present to an emergency room, non-White patients and those with a lower socioeconomic status have been shown to have decreased odds of undergoing surgery.^6,40^

Current studies evaluating socioeconomic factors for shoulder instability are limited to the adult population or surgical patients.^6,20,21,40^ Additionally, many of these studies have used a singular numeric index to measure disparities based on ZIP codes. However, a single value to measure disparities is not specific enough to understand how certain patients may be disadvantaged. Meanwhile, ZIP codes may misrepresent the multiple neighborhoods within them, and census blocks are smaller and more accurate.^26,27^ The Child Opportunity Index (COI) is a composite child-specific index of neighborhood opportunity based on census block data and targets 3 specific disparity areas: educational, health/environmental, and social/economic.

The purpose of this study was to evaluate how educational, health/environmental, and social/economic disparities; race; and insurance status influence the timing of shoulder instability treatment and shoulder instability disease severity among adolescents. We hypothesized that patients who live in neighborhoods with lower educational, health/environmental, and social/economic opportunities would be non-White, have public insurance, be associated with longer times from injury to care, and have more severe shoulder instability lesions.

## Methods

This study was approved by our institutional review board (STU-2020-0977) with a waiver of informed consent.

### Patients

A retrospective review of patient records for shoulder instability was conducted at a single institution specializing in pediatric sports medicine from January 2022 to April 2024. The institution’s electronic medical record was queried for all patients who had a diagnosis indicative of shoulder instability (International Classification of Diseases, 10th Revision codes S43.0 and S43.3) as well as all patients who underwent surgery for shoulder instability (Current Procedural Terminology codes 29806, 23455, 23460, 23465, 23462, or 23466). There were 331 patients evaluated for shoulder instability aged between 10 and 19 years. Patients were excluded if they had another major concomitant injury (n = 10), had a seizure or syndromic disorder (n = 28), did not have advanced imaging available (n = 101), or were missing significant information regarding their initial injury and presentation to a previous clinic (n = 11). Our patient cohort consisted of patients who were treated operatively and nonoperatively.

### Data Collection

Demographic and clinical data were collected, including date of injury, initial physician presentation, and initial orthopaedic evaluation. The date of injury was defined as the date on which the patient reported the instability event that corresponded to the reason why they were seeking an orthopaedic evaluation. Initial physician presentation included emergency department visits or visits to primary care physicians. Similarly, initial orthopaedic evaluation included any evaluation by an orthopaedic surgeon either at an outside clinic or at our institution. The number of previous dislocations that each patient suffered was dichotomized as 1 previous dislocation versus ≥2 previous dislocations.

### Disparity Index

Disparity was measured with the COI, a publicly available validated measure of neighborhood conditions and access to resources that affect child development. The COI 3.0 comprises data from 2012 to 2021, with neighborhoods that are divided based on a census block level. It is composed of 44 indicators organized into 3 domains: educational, health/environmental, and social/economic. The educational domain encompasses school enrollment, standardized test scores, advanced course enrollment, graduation rates, college enrollment, school poverty, adult education attainment, and teacher experience. The health/environmental domain comprises pollution levels, healthy food availability, heat exposure, green space, walkability, and community safety. The social/economic domain includes income levels, housing resources, local nonprofit organizations, home values, employment rates, public assistance, and homeownership. COI categories are classified as very low, low, moderate, high, and very high in order of least opportunity to most opportunity. Patients living in neighborhoods with high opportunity in one domain can still have very low opportunity in another domain. Patient home addresses were collected on intake forms and input into the COI census block locator, which provides the opportunity category level of each domain and subdomain for that census block.^9^ Multiple validation analyses for the COI 3.0 have been performed by including data from the COI 2.0, Social Vulnerability Index, Area Deprivation Index, Opportunity Atlas, United States Census Bureau, and United States Centers for Disease Control and Prevention.^31^

### Imaging

Magnetic resonance imaging (MRI) was used to measure glenoid and humeral bone loss. All MRI examinations were conducted within 6 months of the injury. Measurements of glenoid bone loss were performed using the best-fit circle method and calculated as a percentage of the diameter of the glenoid.^17^ The Hill-Sachs interval (HSI) was measured as the distance from the medial margin of the Hill-Sachs lesion to the rotator cuff insertion on the axial view.^18^ The distance to dislocation (DTD) was calculated for on-track lesions as glenoid track size minus HSI size. For on-track lesions, shoulders were categorized into those with a DTD <10 mm and those with a DTD ≥10 mm. A DTD <10 mm has been shown to be the threshold for an exponentially higher risk of recurrent dislocations.^5^

### Statistical Analysis

COI categories were analyzed as ordinal variables. Continuous dependent variables were analyzed using the Spearman correlation coefficient when compared with the COI, while the Mann-Whitney *U* test was performed when continuous dependent variables were compared with race/ethnicity or insurance status. For categorical variables, the chi-square test was performed with additional secondary binary logistic regression analysis for statistically significant results that controlled for age, race/ethnicity, and insurance status. Patients who were Black, Hispanic, and Asian were also analyzed as non-White. Additional subanalysis was performed comparing younger adolescents (10-14 years) to older adolescents (15-19 years). All statistical analyses were performed using SAS (Version 9.4; SAS Institute), with significance set at *P* < .05.

## Results

Of the 181 patients who met inclusion criteria, 45% were White, 28% were Black, 23% were Hispanic, and 4% were Asian. Additionally, 81% of patients were male, and 19% were female (Table 1). The mean age at the time of injury was 15.8 ± 1.6 years. There were 94% of patients who had anterior shoulder instability, while 6% had posterior shoulder instability. A shoulder dislocation occurred in 85% of patients, while 15% of patients only had a shoulder subluxation. The mechanism of injury was traumatic in 96% of patients and atraumatic in 4% of patients. There were 81% of patients who injured themselves during a sporting event. Of these sporting injuries, 78% occurred from contact sports including football, hockey, boxing, bull riding, and lacrosse. Noncontact sports included tennis, soccer, cheer, and golf. Nonsporting injuries include falls, roughhousing, or motor vehicle accidents. Surgery was performed in 56% of all patients.

**Table 1:** Patient Characteristics*^a^*

		COI Category				
	All (n = 181)	Very Low (n = 27)	Low (n = 21)	Medium (n = 29)	High (n = 40)	Very High (n = 64)
Sex						
Male	143 (79)	25 (93)	13 (62)	24 (83)	33 (83)	48 (75)
Female	38 (21)	2 (7)	8 (38)	5 (17)	7 (18)	16 (25)
Race/ethnicity						
White	81 (45)	3 (11)	10 (48)	11 (38)	22 (55)	35 (55)
Black	51 (28)	14 (52)	3 (14)	8 (28)	9 (23)	17 (27)
Hispanic	41 (23)	10 (37)	7 (33)	9 (31)	8 (20)	7 (11)
Asian	8 (4)	00 (0)	1 (5)	1 (3)	1 (3)	5 (8)

a Data are expressed as n (%). COI, Child Opportunity Index.

### Time From Injury to Initial Presentation

A longer time from injury to initial presentation was associated with a lower overall COI (*r* = −0.15; *P* = .048) (Table 2) and lower educational COI (*r* = −0.19; *P* = .012) (Table 3) but not health/environmental COI (Table 4) or social/economic COI (Table 5). Additionally, a longer time from injury to initial presentation was associated with non-White patients compared with White patients (94 vs 44 days, respectively; *P* = .027) (Table 6) and public insurance compared with commercial insurance (104 vs 55 days, respectively; *P* = .048) (Table 7). There was no difference in the time from injury to initial presentation between younger and older patients. The mean number of days from injury to initial presentation for children in the very low opportunity group was 116 days, whereas for the very high opportunity group, it was 55 days (Table 2).

**Table 2:** Outcomes for Overall COI*^a^*

	Very Low (n = 27)	Low (n = 21)	Medium (n = 29)	High (n = 40)	Very High (n = 64)	*P* Value
Time from injury to initial presentation, d	116.3 ± 221.9	117.9 ± 226.8	53.9 ± 76.2	56.1 ± 102.3	55.4 ± 156.4	**.048**
Time from injury to orthopaedic evaluation, d	137.6 ± 221.2	121.1 ± 226.6	62.4 ± 74.2	68.9 ± 108.1	62.0 ± 157.4	**.027**
Time from injury to MRI, d	153.6 ± 222.0	153.0 ± 240.4	125.8 ± 172.1	78.7 ± 108.3	86.9 ± 169.0	**.033**
Time from injury to surgery, d	192.5 ± 112.4	136.0 ± 113.1	195.3 ± 96.4	113.8 ± 77.5	189.4 ± 195.1	.854
Recurrent dislocations	55.6	23.8	20.7	25.0	26.6	**.041**
Glenoid bone loss, %	8.7 ± 9.7	3.8 ± 7.5	7.4 ± 10.7	4.5 ± 7.3	5.2 ± 8.6	.246
HSI, mm	12.5 ± 6.9	8.3 ± 6.6	9.9 ± 7.8	10.3 ± 6.3	8.2 ± 6.4	.052
DTD <10 mm	75.0	47.6	41.7	52.6	29.0	**.003**
Off-track lesion	11.1	0.0	17.2	5.0	4.7	.125

a Data are expressed as mean ± SD or %. Statistical significance set as *P* < .05 (bold). COI, Child Opportunity Index; DTD, distance to dislocation; HSI, Hill-Sachs interval; MRI, magnetic resonance imaging.

**Table 3:** Outcomes for Educational COI*^a^*

	Very Low (n = 13)	Low (n = 30)	Medium (n = 33)	High (n = 36)	Very High (n = 69)	*P* Value
Time from injury to initial presentation, d	187.5 ± 301.5	102.0 ± 194.5	58.8 ± 78.7	48.9 ± 103.0	54.6 ± 151.0	**.012**
Time from injury to orthopaedic evaluation, d	218.1 ± 294.8	110.3 ± 193.3	66.1 ± 76.3	62.1 ± 110.8	61.3 ± 151.9	**.005**
Time from injury to MRI, d	228.2 ± 296.6	125.7 ± 199.5	106.3 ± 115.0	100.4 ± 170.1	84.9 ± 163.2	**.022**
Time from injury to surgery, d	207.6 ± 85.7	167.9 ± 115.7	185.9 ± 110.1	124.7 ± 68.1	174.7 ± 192.4	.594
Recurrent dislocations	69.2	36.7	15.2	25.0	27.5	**.009**
Glenoid bone loss, %	8.9 ± 10.3	5.6 ± 8.1	5.3 ± 8.1	4.9 ± 9.8	5.7 ± 8.7	.496
HSI, mm	12.8 ± 7.9	9.2 ± 6.7	9.5 ± 7.5	10.8 ± 6.4	8.8 ± 6.4	.209
DTD <10 mm	70.0	60.0	40.0	51.5	33.3	**.045**
Off-track lesion	23.1	0.0	9.1	8.3	5.8	.092

a Data are expressed as mean ± SD or %. Statistical significance set as *P* < .05 (bold). COI, Child Opportunity Index; DTD, distance to dislocation; HSI, Hill-Sachs interval; MRI, magnetic resonance imaging.

**Table 4:** Outcomes for Health/Environmental COI*^a^*

	Very Low (n = 70)	Low (n = 61)	Medium (n = 39)	High (n = 9)	Very High (n = 2)	*P* Value
Time from injury to initial presentation, d	95.7 ± 188.8	50.4 ± 94.9	77.6 ± 194.9	18.3 ± 25.5	3.0 ± 2.8	.164
Time from injury to orthopaedic evaluation, d	108.4 ± 188.2	62.7 ± 102.5	81.8 ± 193.5	20.2 ± 24.7	3.0 ± 2.8	.092
Time from injury to MRI, d	146.6 ± 212.9	83.3 ± 111.7	94.2 ± 201.3	75.1 ± 110.7	11.5 ± 9.2	.052
Time from injury to surgery, d	178.2 ± 108.7	182.8 ± 188.8	124.8 ± 77.2	91.8 ± 54.3	—*^b^*	.051
Recurrent dislocations	32.9	26.2	30.8	11.1	50.0	.580
Glenoid bone loss, %	7.1 ± 9.9	4.8 ± 7.2	5.1 ± 9.2	3.5 ± 8.2	0.0 ± 0.0	.070
HSI, mm	11.3 ± 7.1	8.8 ± 6.5	8.7 ± 6.7	7.8 ± 4.7	6.3 ± 8.9	**.018**
DTD <10 mm	61.3	39.0	35.1	11.1	50.0	**.006**
Off-track lesion	11.4	3.3	7.7	0.0	0.0	.412

a Data are expressed as mean ± SD or %. Statistical significance set as *P* < .05 (bold). COI, Child Opportunity Index; DTD, distance to dislocation; HSI, Hill-Sachs interval; MRI, magnetic resonance imaging.

b No patients underwent surgery.

**Table 5:** Outcomes for Social/Economic COI*^a^*

	Very Low (n = 18)	Low (n = 25)	Medium (n = 26)	High (n = 45)	Very High (n = 67)	*P* Value
Time from injury to initial presentation, d	107.8 ± 195.1	127.8 ± 256.4	66.6 ± 94.7	51.6 ± 97.9	56.4 ± 153.1	.051
Time from injury to orthopaedic evaluation, d	134.7 ± 196.9	133.8 ± 255.0	75.5 ± 91.9	62.3 ± 104.0	63.5 ± 153.9	**.025**
Time from injury to MRI, d	154.0 ± 198.1	163.5 ± 260.7	133.5 ± 187.2	88.4 ± 115.0	80.7 ± 162.3	**.020**
Time from injury to surgery, d	197.0 ± 130.5	159.9 ± 101.9	195.8 ± 83.1	122.8 ± 92.1	181.9 ± 190.7	.745
Recurrent dislocations	55.6	36.0	26.9	17.8	28.4	**.049**
Glenoid bone loss, %	6.3 ± 9.4	7.4 ± 9.3	6.1 ± 10.3	4.7 ± 7.8	5.3 ± 8.6	.079
HSI, mm	12.0 ± 7.5	11.4 ± 6.0	9.1 ± 8.3	10.1 ± 6.3	8.3 ± 6.4	**.020**
DTD <10 mm	68.8	70.8	36.4	50.0	29.2	**.001**
Off-track lesion	11.1	4.0	15.4	6.7	4.5	.353

a Data are expressed as mean ± SD or %. Statistical significance set as *P* < .05 (bold). COI, Child Opportunity Index; DTD, distance to dislocation; HSI, Hill-Sachs interval; MRI, magnetic resonance imaging.

**Table 6:** Outcomes by Race*^a^*

	White	Non-White	*P* Value
Time from injury to initial presentation, d	44.2 ± 104.8	93.9 ± 189.8	**.027**
Time from injury to orthopaedic evaluation, d	50.4 ± 104.0	107.1 ± 191.2	**.012**
Time from injury to MRI, d	71.4 ± 117.2	139.4 ± 210.5	**.007**
Time from injury to surgery, d	136.8 ± 99.7	187.9 ± 155.6	.083
Recurrent dislocations	16.1	40.0	**<.001**
Glenoid bone loss, %	3.9 ± 6.8	7.1 ± 10.0	**.013**
HSI, mm	8.4 ± 7.1	10.7 ± 6.4	**.022**
DTD <10 mm	29.9	56.6	**<.001**
Off-track lesion	4.9	9.0	.390

a Data are expressed as mean ± SD or %. Statistical significance set as *P* < .05 (bold). COI, Child Opportunity Index; DTD, distance to dislocation; HSI, Hill-Sachs interval; MRI, magnetic resonance imaging.

**Table 7:** Outcomes by Insurance Status*^a^*

	Commercial	Public	*P* Value
Time from injury to initial presentation, d	55.0 ± 136.7	104.4 ± 192.9	**.048**
Time from injury to orthopaedic evaluation, d	65.1 ± 140.1	114.3 ± 191.3	.507
Time from injury to MRI, d	85.5 ± 146.7	155.1 ± 221.2	**.028**
Time from injury to surgery, d	155.0 ± 120.0	192.1 ± 166.4	.221
Recurrent dislocations	23.3	41.0	**.014**
Glenoid bone loss, %	4.5 ± 7.8	7.4 ± 10.3	.057
HSI, mm	9.5 ± 6.6	10.0 ± 7.2	.603
DTD <10 mm	42.1	50.9	.281
Off-track lesion	5.8	9.8	.324

a Data are expressed as mean ± SD or %. Statistical significance set as *P* < .05 (bold). COI, Child Opportunity Index; DTD, distance to dislocation; HSI, Hill-Sachs interval; MRI, magnetic resonance imaging.

### Time From Injury to Orthopaedic Evaluation

A longer time from injury to orthopaedic evaluation was associated with a lower overall COI (*r* = −0.17; *P* = .027) (Table 2), lower educational COI (*r* = −0.21; *P* = .005) (Table 3), lower social/economic COI (*r* = −0.17; *P* = .025) (Table 5), and non-White patients compared with White patients (107 vs 50 days, respectively; *P* = .012) (Table 6). There was no difference in the time from injury to orthopaedic evaluation between younger and older patients. The mean number of days from injury to orthopaedic presentation for children in the very low opportunity group was 138 days, whereas for the very high opportunity group, it was 62 days (Table 2).

### Time From Injury to MRI

A longer time from injury to MRI was associated with a lower overall COI (*r* = −0.16; *P* = .033) (Table 2), lower educational COI (*r* = −0.17; *P* = .022) (Table 3), lower social/economic COI (*r* = −0.18; *P* = .020) (Table 5), non-White patients compared with White patients (139 vs 71 days, respectively; *P* = .007) (Table 6), and public insurance compared with commercial insurance (155 vs 86 days, respectively; *P* = .028) (Table 7). There was no difference in the time from injury to MRI between younger and older patients. The mean number of days from injury to MRI for children in the very low opportunity group was 154 days, whereas for the very high opportunity group, it was 87 days (Table 2).

### Time From Injury to Surgery

There was no association between the time from injury to surgery and any of the COI domains, non-White race/ethnicity, insurance status, or age. The mean number of days from injury to surgery for children in the very low opportunity group was 193 days, whereas for the very high opportunity group, it was 189 days (Table 2).

### Recurrent Dislocations

A history of recurrent dislocations was associated with a lower overall COI (B = −3.27; *P* = .041) (Table 2), lower educational COI (B = −3.01; *P* = .009) (Table 3), and lower social/economic COI (B = −3.65; *P* = .049) (Table 5). Non-White patients were associated with more recurrent dislocations compared with White patients (40% vs 16%, respectively; *P* < .001) (Table 6). Specifically, 51% of Black patients and 32% of Hispanic patients had previous recurrent dislocations, in contrast to 16% of White patients. Having public insurance was associated with more recurrent dislocations compared with commercial insurance (41% vs 23%, respectively; *P* = .014) (Table 7). There was no difference in the history of recurrent dislocations between younger and older patients.

### Glenoid Bone Loss

Non-White patients had significantly greater glenoid bone loss than White patients (7% vs 4%, respectively; *P* = .013) (Table 6). There was no association between glenoid bone loss and any of the COI domains, insurance status, or age.

### HSI Size

A larger HSI was associated with a lower health/environmental COI (*r* = −0.18; *P* = .018) (Table 4) and lower social/economic COI (*r* = −0.17; *P* = .020) (Table 5). Non-White patients had a significantly larger HSI than White patients (11 vs 8 mm, respectively; *P* = .022) (Table 6). There was no association between HSI size and insurance status. Older adolescents were associated with more Hill-Sachs lesions than their younger counterparts (78% vs 57%, respectively; *P* = .006).

### DTD <10 mm for On-Track Lesions

Patients with a DTD <10 mm were associated with a lower overall COI (B = −7.59; *P* = .003) (Table 2), lower educational COI (B = −8.38; *P* = .045) (Table 3), lower health/environmental COI (B = −7.88; *P* = .006) (Table 4), and lower social/economic COI (B = −8.22; *P* = .001) (Table 5). Non-White patients were associated with a higher incidence of a DTD <10 mm compared with White patients (57% vs 30%, respectively; *P* < .001) (Table 6). Insurance status and age were not associated with a DTD <10 mm.

### Off-Track Lesions

There was no association between the presence of an off-track lesion and any of the COI domains, race/ethnicity, or insurance status. Older adolescents were associated with more off-track lesions than their younger counterparts (9% vs 0%, respectively; *P* = .038).

## Discussion

In our study, we employed a composite index of childhood neighborhood opportunity to evaluate how disparities are associated with the timing of care and shoulder instability severity in adolescents treated both operatively and nonoperatively. Using specific disparity domains can help public health policies by targeting particular areas to improve patient outcomes for those from disadvantaged neighborhoods, who we found to be at a higher risk for recurrent shoulder dislocations.^1^ Timely treatment of severe lesions can help to prevent further dislocations and reduce the fear of reinjuries, thus allowing adolescents to return to activities and socialize with their peers.^13,24,25,35^

Important steps for the appropriate care of a shoulder instability episode are evaluations by orthopaedic surgeons and diagnostic imaging. Timely access to these is paramount, as delays have effects on intervention success, recovery time, and instability recurrence.^2^ We found that patients living in neighborhoods with lower opportunities, specifically educational and social/economic opportunities, took longer to present to an orthopaedic surgeon and to undergo shoulder MRI. A lack of educational opportunities can be indicative of poor health literacy, which may be the reason for not following up with an orthopaedic surgeon after shoulder reduction, not returning for follow-up appointments, or incomplete physical therapy regimens. Socioeconomic barriers may include transportation issues, inability to miss work for appointments, conflicting priorities with child care, and type of health insurance, which can all lead to delays in adequate shoulder instability care.

Additionally, non-White patients and those with public insurance also had longer times from the initial shoulder dislocation to medical care compared with White patients and those with commercial insurance, respectively. This is consistent with a study by Hung et al,^21^ who examined insurance status for pediatric shoulder instability outcomes. They found that privately insured patients were seen 5 times faster and underwent MRI 4 times faster than publicly insured patients.^21^ Similarly, Patel et al^33^ examined socioeconomic factors among pediatric anterior cruciate ligament (ACL) injuries and found that publicly insured patients had greater delays in the time from injury to orthopaedic evaluation, to MRI, and to surgery. However, in contrast to Patel et al’s^33^ study, we did not find an association in the time from injury to surgery for our patients who were publicly insured.^21^ Differences in the ideal timing of ACL reconstruction and shoulder instability surgery could be a reason. Unlike shoulder instability surgery, before ACL reconstruction, patients often need to regain full knee range of motion, which may lead to delays in surgery if preoperative therapy is not performed. Additional factors for surgical treatment timing may include patient age, sports played, and timing of the dislocation relative to the sporting season. These factors as they relate to the timing of surgery should be investigated in future studies.

Patients with lower educational and social/economic opportunities, non-White patients, and those with public health insurance were associated with multiple shoulder dislocations before the initial presentation. This may be related to the educational and social/economic barriers of obtaining timely care after an initial dislocation event, as previously described. Our findings are consistent with a study by Hettrich et al^20^ examining adolescents and adults undergoing shoulder instability surgery. They found that a greater percentage of non-White patients had at least 2 previous shoulder dislocations compared with White patients.^20^ This corresponded with more frequent articular cartilage lesions, glenoid bone loss, and Hill-Sachs lesions.^20^ In Hung et al’s^21^ study, publicly insured patients also had a higher incidence of multiple previous dislocations. With delays in care, patients may experience further shoulder dislocations before adequate treatment by a primary care physician or orthopaedic surgeon, which can lead to secondary associated articular cartilage lesions, glenoid and humeral bone loss, pain, and loss of daily function.^8,11,16,29^ Importantly, adolescent mental health may be impacted, as delays in care may lead to a longer time away from sports and social isolation.^7,32^ For patients whose identity is strongly tied to sports, the mental health effect of not being able to play sports is magnified.^2^

Regarding the severity of shoulder instability, we found that a DTD <10 mm for on-track lesions was associated with worse childhood opportunity in all 3 educational, health/environmental, and social/economic domains along with non-White patients. The DTD is calculated based on the remaining glenoid track and HSI in which 10 mm is the threshold for an exponentially higher risk of recurrent shoulder dislocations.^5^ Additionally, we found that non-White patients had greater glenoid bone loss and a larger HSI. Hettrich et al^20^ similarly found that non-White patients had increased rates of glenoid bone loss >10% and the presence of Hill-Sachs lesions. The severity of shoulder instability lesions upon an orthopaedic evaluation has a consequential effect on treatment algorithms. Patients already with significant bone loss or a high risk of recurrence have worse prognoses and surgical outcomes.^14^ They may not be afforded the opportunity of observation and nonoperative management. Appropriate surgical treatment to stabilize larger lesions may require more invasive procedures, such as the Latarjet or remplissage procedure. Previous studies have found that after arthroscopic Bankart repair, the most common reasons for athletes not returning to play were a fear of reinjuries in as high as 44% and persistent instability.^24,35,42^ This fear of reinjuries, or kinesiophobia, has been associated with depression and worse quality of life in patients with shoulder instability.^7,43^ Weekes et al^43^ reported that 50% of patients with shoulder instability had clinical depression among surgical patients preoperatively. Therefore, barriers associated with more severe shoulder instability lesions with a higher risk of recurrence are imperative to address to reduce persistent instability and the potential effects on mental health.

Strategies to improve care for vulnerable patients with shoulder instability include improved screening, specialized treatment protocols, and community outreach.^39^ Screening may be built into electronic medical record systems to identify patients living in low-opportunity neighborhoods.^36^ Identification can allow the practitioner to follow specialized nonoperative or postoperative protocols to achieve equitable care.^28^ Additional services such as social work can help to identify affordable physical therapists close to home. Patient-tailored education can help to ensure that patients with low health literacy understand their diagnosis and prognosis.^15,30^ Finally, community outreach initiatives can include collaborating with local schools or sports leagues in low-opportunity neighborhoods to educate athletes on shoulder instability and sports-related injuries as well as identify high-risk athletes.^3,23^ Specific data and maps of low-opportunity neighborhoods for each United States city are readily available on the Diversity Data Kids website.^9^

### Limitations

This study is not without its limitations. This is a retrospective study from a single institution that only focused on pediatric orthopaedic sports injuries, which may influence the generalizability of the study to other clinical settings. We were unable to ascertain the exact reasons why some patients presented in a delayed fashion to either a primary care provider, another orthopaedic specialist, or our institution. Accordingly, about a quarter of the patients had undergone previous evaluations with other orthopaedic surgeons before presentation at our institution as a second opinion. Additionally, we did not find the COI to be associated with glenoid bone loss or off-track lesions, which may be because of our relatively low incidence of patients with recurrent dislocations before presentation at 29%. Finally, patients without advanced imaging were excluded from this study because of the inability to accurately measure bone loss, and these patients may have come from various socioeconomic backgrounds. The strengths of this study are that our institution is a tertiary care center with a broad referral base in a large metropolitan area and accepts patient regardless of their ability to pay, which provides a well-represented portrayal of the community. The institution’s financial structure allowed our study to evaluate the effect of insurance status on shoulder instability outcomes without its influence on the timing of care once patients presented to our institution. Our cohort also included a broad representation of races and ethnicities, the majority of whom were not White. Importantly, unlike other studies, this study included nonsurgical patients and evaluated disparities based on smaller census block neighborhoods rather than ZIP codes.

## Conclusion

Children living in neighborhoods with fewer educational and social/economic opportunities were associated with longer times from injury to medical care for shoulder instability and were at a higher risk for recurrent shoulder dislocations. This study can help others to target specific domains to improve the shoulder instability outcomes of children living in low-opportunity neighborhoods by expanding resources through legislation, outreach, and specialized care. A better understanding of the barriers that are presented to our patients helps us to provide higher quality care and can hopefully improve outcomes for all our patients.
